# Cost-effective, portable, patient-dedicated three-dimensional automated breast ultrasound for point-of-care breast cancer screening

**DOI:** 10.1038/s41598-023-41424-7

**Published:** 2023-09-01

**Authors:** Claire Keun Sun Park, Tiana Trumpour, Amal Aziz, Jeffrey Scott Bax, David Tessier, Lori Gardi, Aaron Fenster

**Affiliations:** 1 Department of Medical Biophysics, Schulich School of Medicine and Dentistry, Western University, London, ON N6A 3K7 Canada; 2 Robarts Research Institute, 1151 Richmond St. N., London, ON N6A 5B7 Canada; 3 School of Biomedical Engineering, Faculty of Engineering, Western University, London, ON N6A 3K7 Canada; 4 Division of Imaging Sciences, Department of Medical Imaging, Schulich School of Medicine and Dentistry, Western University, London, ON N6A 3K7 Canada

**Keywords:** Biomedical engineering, Breast cancer, Cancer screening

## Abstract

Breast cancer screening has substantially reduced mortality across screening populations. However, a clinical need persists for more accessible, cost-effective, and robust approaches for increased-risk and diverse patient populations, especially those with dense breasts where screening mammography is suboptimal. We developed and validated a cost-effective, portable, patient-dedicated three-dimensional (3D) automated breast ultrasound (ABUS) system for point-of-care breast cancer screening. The 3D ABUS system contains a wearable, rapid-prototype 3D-printed dam assembly, a compression assembly, and a computer-driven 3DUS scanner, adaptable to any commercially available US machine and transducer. Acquisition is operator-agnostic, involves a 40-second scan time, and provides multiplanar 3D visualization for whole-breast assessment. Geometric reconstruction accuracy was evaluated with a 3D grid phantom and tissue-mimicking breast phantoms, demonstrating linear measurement and volumetric reconstruction errors < 0.2 mm and < 3%, respectively. The system’s capability was demonstrated in a healthy male volunteer and two healthy female volunteers, representing diverse patient geometries and breast sizes. The system enables comfortable ultrasonic coupling and tissue stabilization, with adjustable compression to improve image quality while alleviating discomfort. Moreover, the system effectively mitigates breathing and motion, since its assembly affixes directly onto the patient. While future studies are still required to evaluate the impact on current clinical practices and workflow, the 3D ABUS system shows potential for adoption as an alternative, cost-effective, dedicated point-of-care breast cancer screening approach for increased-risk populations and limited-resource settings.

## Introduction

Globally, breast cancer is the most commonly diagnosed cancer in women, with over 2.3 million women newly diagnosed and 650,000 deaths in 2020^1^. While breast cancer has a high incidence, it has been shown to have a relatively favorable survival rate, which can be attributed to the detection of early-stage breast cancers through screening in predominantly developed countries^2–4^. Mammography is undoubtedly an important factor in reducing breast cancer-related mortality, notably, in screening populations over 50 years of age and those assigned female at birth^5^. However, population-based screening protocols and recommendation guidelines differ widely across population age, frequency, and modalities^6^. Beyond screening average-risk populations, there exist several other groups and risk-factors that increase the overall risk for developing breast cancer. These specific risks broadly include hereditary breast cancer, with inherited BRCA1 or BRCA2 gene mutations, a personal or family history of breast cancer, a history of chest radiation, and dense breasts^7, 8^. Moreover, younger women are more likely to develop more aggressive breast cancers, which often present at more advanced stages at the time of detection and diagnosis^9–11^. Across these intermediate to high-risk populations, current breast cancer screening recommendations and protocols lack complete consensus and may be suboptimal^7, 12^.

In women with dense breasts, which refers to an increased proportion of fibroglandular tissues compared with fatty adipose tissues, the mammographic sensitivity to detect breast cancer is substantially reduced. Increased breast density has a masking effect in mammograms, due to challenges in discriminating between dense breast tissues and malignancies^8^. This limitation may potentially result in delayed diagnosis between recommended screens and interval cancers, and poorer overall prognosis in these women with dense breasts^13^. Moreover, breast density alone is a strong, independent risk-factor for the development of breast cancer^13^. While in North America, the proportion of women with dense breasts represents approximately 40% of all screening population^14^, the proportion is substantially higher amongst diverse ethnic and racial populations^15, 16^. Due to the aforementioned challenges in screening increased-risk women with dense breasts, supplemental screening with magnetic resonance imaging (MRI), digital breast tomosynthesis (DBT), and ultrasound (US) have been effectively used to improve early detection of breast cancer^17, 18^. Despite its clinical utility and potential diagnostic advantages, the value of supplemental screening with these modalities remains unclear^7^. Furthermore, the required facilities, specialized healthcare operators and infrastructure needs, associated costs, and contraindications limit their widespread use as accessible screening modalities, especially in limited-resource settings.

Beyond established risk-factors, there are clear disparities in breast cancer screening in several equity-seeking and marginalized populations, resulting in a disproportionate increase in cancer-related morbidities. While the term ‘women’ has been widely used in clinical practice and literature, as those who are assigned female at birth, other sexual and gender minorities, such as transgender or nonbinary individuals, may have similar associated and increased risk-factors as cisgender women. In sexual and gender minorities, evidence-based recommended cancer screening guidelines are unclear^19^, and preventative health behavior, including breast cancer screening, is substantially lower due to barriers to care^20^. Specifically, there exists an elevated risk for developing breast cancer in these minority populations, compared with their heterosexual counterparts^21^. These individuals and groups are less likely to participate in recommended screening, as they often lack equitable and inclusive practices, which may ultimately result in diagnosis of breast cancer at more advanced stages, where intervention and treatments are less successful^20–22^.

Cancer-related health inequities stem from structural marginalization in race and ethnicity, sexual orientation and gender, and other social determinants of health, including socioeconomic status and environmental factors (i.e., limited-resource, rural or remote areas) that limit accessibility, impose cost constraints and reduce inclusivity of these individuals and groups in cancer care. For many of these populations, point-of-care technologies can provide a practical solution by allowing for data acquisition at the site of care and actionable information for rapid clinical decision-making, alleviating challenges associated with limited-resource or cost-constrained healthcare settings. Therefore, the development of more accessible, cost-effective, and point-of-care technologies for early-detection and diagnosis in increased-risk populations has the potential to advance breast cancer-related health equities and outcomes.

Ultrasound (US) is a widely available, non-ionizing, compact, and cost-effective imaging modality in clinical practice^23^. With increased-risk populations, specifically those with dense breasts, handheld (HH) US has been shown to be an effective supplemental screening method for detecting small, early-stage, and invasive breast cancers^18, 24–26^. However, HHUS is operator-dependent and time-consuming, which limit its use as a widespread screening modality. Automated breast (AB) US is an emerging method that shows utility for supplemental screening in women with dense breasts^27^. ABUS imaging allows for more standardized acquisition, improved operator-dependence, and reproducibility with three-dimensional (3D) volumetric whole-breast assessment^28, 29^. Most common, commercially available ABUS systems employed in clinical practice are the Invenia ABUS (GE Healthcare, Chicago, Illinois, United States) and ACUSON S2000 Automated Breast Volume Scanner (ABVS) (Siemens Healthineers, Munich, Germany). These ABUS systems involve a supine acquisition approach and are comprised of an external articulated manipulator, ultrawide high-frequency 5–14 MHz US transducer, touchscreen monitor (scan station) and workstation (view station) for 3D image reconstruction, visualization, and interpretation. The ABUS image acquisition involves a computer-driven mechanical scan to automatically translate the ultrawide US transducer within a sonolucent compression paddle, with a disposable membrane for coupling. Acquisition typically involves three views: anteroposterior (AP), lateral (LAT), and medial (MED) views in each breast, with additional image views acquired to expand the volumetric coverage in larger breasts, which takes approximately a 10-min scan time^27^.

While currently available 3D ABUS approaches have shown clear clinical utility and high diagnostic performance for supplemental screening, especially in women with dense breasts^27, 30^, there are several limitations. High quality ABUS requires operator training for proper patient positioning and ultrasonic coupling, in order to avoid artifacts during image acquisition^31, 32^. Moreover, the US transducer paddle is not designed to fit all women and their breasts, nor is adaptable to different patient populations^30^. The ABUS acquisition has also been reported as painful by some patients due to the required compression from ultrawide US transducers, which are required to obtain reliable images^33^. Although these limitations can be mitigated with increased operator training for patient positioning and careful acquisition protocols, alternative, more robust approaches are still necessary^31, 32^. Beyond these technical limitations, most commercially available ABUS systems are relatively bulky and costly, as they require complete machines with articulated manipulators, costly ultrawide US transducers, or specialized patient examination tables for prone acquisition approaches^28, 34^.

To address these aforementioned challenges, this work aims to develop an alternative, cost-effective, portable, and patient-dedicated 3D ABUS system for point-of-care breast cancer screening with utility in increased-risk populations and limited-resource settings. The proposed 3D ABUS system utilizes a rapid-prototype, dam assembly to conform to the patient’s breast and contain coupling gel for a supine acquisition approach. This paper describes the design and fabrication of the dedicated 3D ABUS system, reports on experimental validation in various test phantoms, and demonstrates the system’s utility in three proof-of-concept healthy volunteers, representing diverse patient geometries and breast sizes. Importantly, the 3D ABUS system can accommodate any commercially available US transducer, utilizes rapid-prototyping, 3D-printing, and economical materials, opening the door to personalized, patient-specific 3D ABUS imaging. The 3D ABUS acquisition approach removes operator-dependence to acquire high-resolution 3DUS images. Moreover, it is capable of point-of-care imaging with real-time multiplanar 3D visualization enabling online or offline whole-breast assessment. Lastly, we demonstrated the 3D ABUS system’s versatility and robustness across various parameters and acquisition conditions, improving ultrasonic coupling, tissue stabilization for image acquisition, and mitigating artifacts due to patient breathing and motion.

## Materials and methods

### Design of the dedicated 3D ABUS system

**Figure 1:**
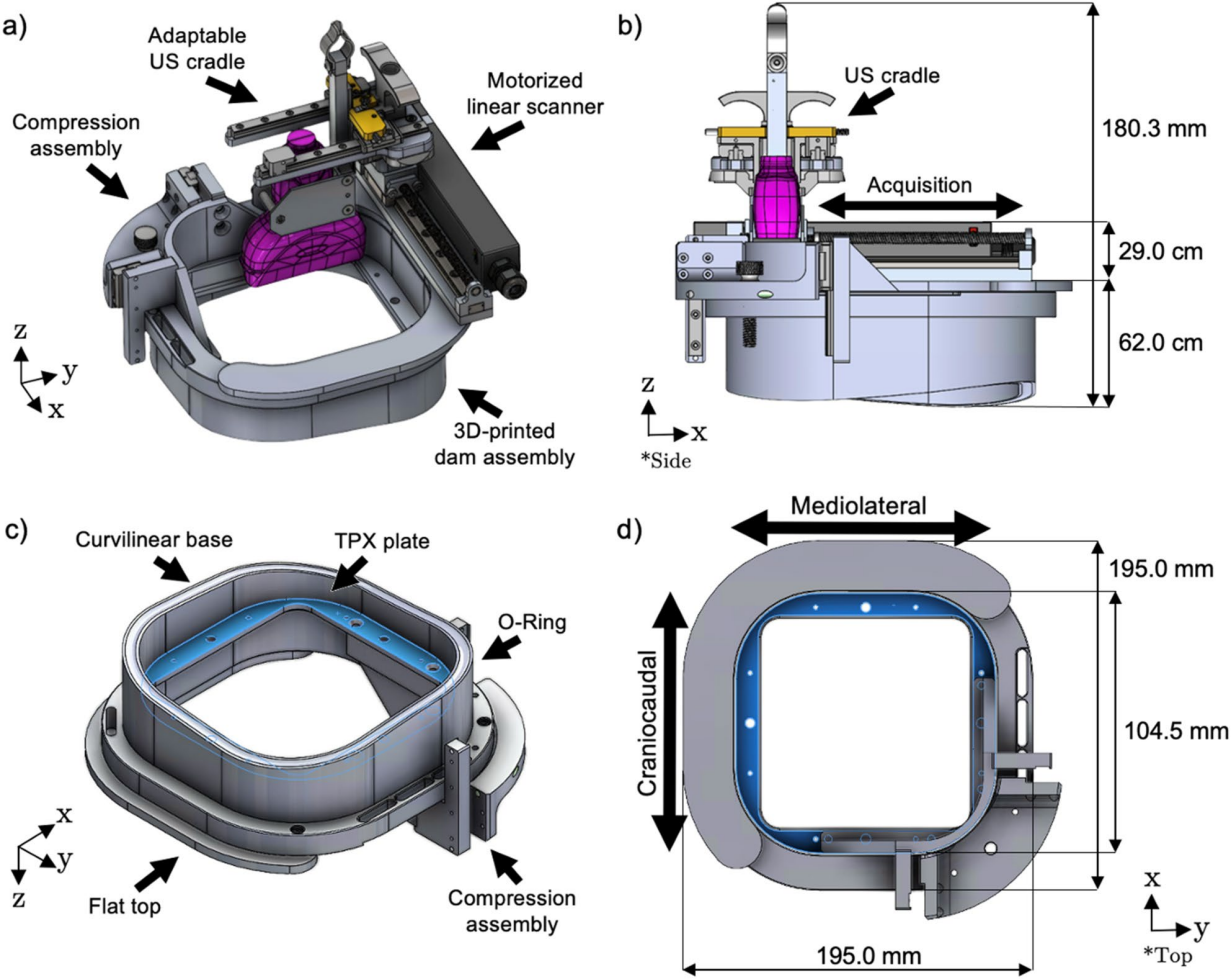
Top Row: Computer-aided design (CAD) of the dedicated 3D ABUS system: (**a**) 3D perspective view, showing the patient-dedicated wearable 3D-printed dam assembly, compression assembly, and motorized 3DUS scanner; and (**b**) side view, illustrating the geometry of the patient-conforming 3D-printed dam assembly and acquisition direction of the US transducer, perpendicular to the 2DUS scan plane. Bottom Row: Wearable, rapid-prototype 3D-printed dam assembly: (**c**) perspective bottom view, showing its components; and (**d**) top view, illustrating the two possible 3DUS configurations (mediolateral and craniocaudal orientations) for 3D ABUS image acquisition. The 3D measurement dimensions of the prototype 3D ABUS system are shown in the side (**b**) and top (**d**) views. The maximum scan distance is 104.5 mm in either craniocaudal or mediolateral acquisition directions.

The dedicated 3D ABUS system, shown in Fig. 1, consists of (a) a wearable assembly made from a rapid-prototype 3D-printed dam, (b) an optional and adjustable compression assembly, and (c) a motorized computer-driven linear scanner for automated 3DUS image acquisition^35^.The 3D ABUS system was fabricated entirely with rapid-prototyping, 3D-printing, and low-cost economical materials, including Delrin, Aluminum, and Brass, commercially available linear bearings and electronic components. The 3D-printed components were fabricated with a 3D Idea Builder 3D40 3D Printer (Dremel, Racine, Wisconsin, United States) and 1.75 mm diameter PLA filament, which were printed with a standard resolution of 0.20 mm. The adaptable US transducer cradle can accommodate any commercially available 2D linear array US transducer from any US machine. The approximate weight of each constituent component is 613 g for the wearable dam assembly, 397 g for the compression assembly, and 500 g for the motorized scanner. The total weight of the prototype 3D ABUS system is 1510 g without the linear US transducer, and approximately 1625 g with an integrated Canon (Canon Medical Systems, Tochigi, Japan) 14L5 (PLT-1005BT) (10 MHz) linear array transducer.

#### Wearable 3D ABUS dam and compression assembly

The dedicated 3D ABUS system is a portable, compact system that is entirely contained on the wearable dam assembly, which serves as the base of the system and is affixed directly onto the patient with adjustable nylon webbings and quick-release buckles. The system’s dam assembly is positioned around breast, with the opening for the scanning assembly accessible from the anterior side of the patient, allowing for supine positioning for image acquisition. The wearable dam is a personalizable 3D-printed component that is shaped like a hollow square with rounded corners, (Fig. 1c) with a flat surface at the top to serve as the base of the 3D ABUS scanner (Fig. 1d) and curvilinear surface at its base to conform to the patient. While the curvilinear base can be personalized and adapted to each patient or specific patient populations, the prototype dam was designed based on a computer-aided design (CAD) 3D human model of a female (SolidWorks, CAD Human Female Poseable, CADHuman.com) that was dynamically scaled to 50th percentile, representing the median weight and height of an American female. The prototype dam was then tested and modified based on feedback from a small focus group of healthy male and female volunteers with variable geometries and breast sizes, ensuring that the design was suitable and met the user requirements. The feedback highlighted aspects of the dam assembly that required adjustment for primarily enhancing comfort and functionality, which are evaluated and discussed in later sections of this work. After modifications were implemented, the healthy volunteers all self-reported that the dam assembly was able to fit comfortably against their chest wall, with no leakage of US gel outside of the dam assembly.

The curvilinear base, as shown in Fig. 1c and d, follows the perimeter of the breast with the main four walls of the square shaped dam positioned on the: (a) medial side parallel to and positioned on the sternum, (b) on the superior border of the breast, (c) inferior border of the breast, and (d) lateral edge of the breast. A skin-safe, soft, and flexible closed-cell blended Neoprene tube (10.0 mm Soft Rubber Tube for Air and Water, McMaster-Carr, Elmhurst, Illinois, United States) was molded into the underside of the curvilinear surface to optimize comfort and enable a more moldable template to conform to the patient. The curvilinear base further allows for a replaceable, skin-safe, and stretchable ultra-thin sonolucent membrane (0.015-inch Silicone Rubber Sheet, McMaster-Carr, Elmhurst, Illinois, United States) to be rapidly attached to the base assembly with a removable Buna-N (NBR) O-Ring, shown in Fig. 1c. When affixed to the patient, the taut membrane allows for stabilization of the patient’s breast and tissues. The length and shape of the curvilinear base can be adjusted through rapid-prototyping to account for diverse breast morphology to optimize coupling between the membrane and chest wall. To allow for superior coupling with the skin, multipurpose Polysonic US lotion (Parker Laboratories, Inc., Fairfield, NJ, United States) is applied to the membrane. In addition, the membrane contains the US transmission gel inside the dam assembly, allowing for a more efficient cleanup by removing the entire dam assembly and disposing of the replaceable membrane containing the US gel.

The dam assembly further supports an optional adjustable compression plate assembly and the motorized 3DUS scanner. The compression plate assembly is mounted on the top surface of the dam assembly and secured with a clamping screw. The compression plate is made from a 10.0 × 10.0 cm TPX polymethylpentene (PMP) (Mitsui Chemicals Inc., Tokyo, Japan) and is supported with a thin aluminum frame, which is positioned above the breast and parallel to the chest wall. The assembly contains an adjustable turn knob mechanism to manually elevate or lower the TPX plate for immobilization or compression of the breast. A light compression would stabilize the breast and eliminate any air gaps or bubbles in the US transmission gel. An increased compression would reduce tissue thickness, without painful application or discomfort, to homogenously distribute the breast tissues and improve image quality by allowing increased US frequency by reducing the imaging depth.

#### 3D ABUS scanner and 3DUS image acquisition

The 3D ABUS scanner consists of a computer-driven motorized-drive mechanism (Faulhaber MICROMO LLC, Clearwater, FL, USA) on a stainless-steel linear stage (THK, Tokyo, Japan) for hands-free, operator-independent, automated 3DUS image acquisition. The linear motor stage employed in our prototype 3D ABUS system is affixed to the patient dam with a positioning accuracy of ± 0.06 mm and allows a maximum travel distance of 104.5 mm for the scan head to translate along the rail in one direction, as shown in Fig. 1b. While the current motor stage and configuration are optimized for the specific dam dimensions, alternative motor stages with an appropriate travel range could be used to ensure that the entire volumetric field-of-view within the dam assembly is covered. The scanner contains an adaptable US cradle, that can accommodate any commercially available linear US transducer. The 3D ABUS scanner assembly allows for three degrees-of-freedom for motion: (a) linear translation for 3DUS image acquisition in the elevational direction of the US beam, (b) lateral movement in the axial direction to expand volumetric coverage, and (c) elevational motion to adjust the US transducer height for optimizing coupling with the breast tissues or TPX compression plate. The system is connected by single Universal Serial Bus (USB) to any portable computer (PC) to interface with an in-house workstation and custom software modules for real-time 3DUS acquisition, multi-image registration and fusion of acquired 3DUS images, and multiplanar 3D visualization. All software modules were written in C++ and maintained in Microsoft Visual Studio 2013 (Visual C++ 12.0, Microsoft Cooperation, Redmond, Washington, United States). Although the 3D ABUS system can accommodate any commercially available US machine and linear US transducer, a Canon Aplio i800 US system (Canon Medical Systems, Tochigi, Japan) and a high-resolution 14L5 (PLT-1005BT) (10 MHz) linear array transducer with a 58-mm footprint width were used in this prototype.

Three-dimensional ABUS image acquisition involves activating the motorized linear scanner to automatically translate the US transducer perpendicular to the 2DUS image plane (i.e., in the US elevational direction) to a specified scan distance, depending on the specific 3D-printed dam assembly window opening and US transducer dimension constraints. During 3DUS image acquisition, spatially encoded 2DUS images are continually acquired at a fixed and adjustable spatial interval (frame density setting, 2DUS frames acquired per mm) as the US transducer is translated along its track. The 2DUS images are acquired into any PC via an Epiphan Digital Visual Interface (DVI) to USB interface to video frame grabber (Epiphan Systems, Inc., Ottawa, Ontario, Canada). As the 2DUS images are collected, a 3DUS image is reconstructed in real-time, providing a 3D ABUS image at the completion of the scan period^36^. Therefore, the 3D spatial resolution of the resultant 3DUS image is a combination of the native in-plane (axial and lateral) resolution of the acquired 2DUS images, and out-of-plane (elevational) resolution, as determined by the native elevational resolution of the US transducer and frame spatial density of the acquired 2DUS images. If the spatial density is sufficiently high, as determined experimentally, the out-of-plane resolution is equivalent to the native elevational resolution of the US transducer.

The removable motorized 3D ABUS scanner can be used in one of two orthogonal configurations: (a) craniocaudal direction to acquire transverse 2DUS images, or (b) mediolateral direction to acquire sagittal 2DUS images (Fig. 1d). The 3DUS scanner can be positioned in either orthogonal configuration is performed using a small set of locating pins and stabilization magnets on the top plate of the dam, with corresponding receiving holes in the removable 3DUS scanner assembly.

#### Dedicated 3D ABUS image reconstruction, multi-image registration and fusion

While the scan distance, in the elevational direction of the US beam, can be adjusted to acquire an increased volumetric region, since the volumetric field-of-view of most conventional linear US transducers are unable to cover the entire breast during a single 3DUS scan, the US cradle, containing the US transducer, can be shifted laterally (axial or perpendicular to its scanning direction) on the mounting rail to another preset position. Once the transducer has been relocated relative to the first 3DUS scan, the motorized scanner is activated again to acquire a second 3DUS image. Since the two 3DUS images are at a known position (distance and orientation) relative to each other, this allowed the software to combine the acquired 3DUS images with a transformation matrix. The combined whole-breast 3DUS image was reconstructed by transforming each 3D voxel position into the image coordinate space, which corresponds to the tracked position and orientation of the acquired images. Two or more parallel and partially overlapping 3DUS images then can be registered and fused in real-time using a rigid registration and weighted voxel-based algorithm (VBA) that assigns the intensity values of overlapping 3D voxels^37^. This VBA determines the intensity value of a fused voxel based on the relative distance to an edge in the overlapped 3D volume. Since the position and orientation of the 3DUS image volumes are known, the weighting factors can be pre-determined to optimize computational speed when fusing multiple 3DUS images and assigning overlapping voxel values. The final combined 3D ABUS image depicts a whole-breast volume that can be viewed and manipulated in any direction and orientation using our in-house 3D multiplanar viewing software.

### 3D ABUS experimental validation methods

Geometric reconstruction accuracy of the acquired 3D ABUS images was verified with both linear and volumetric measurements. For these experiments, the compression assembly and sonolucent membrane were not included on the 3D ABUS system.

#### Linear measurement accuracy

**Figure 2:**
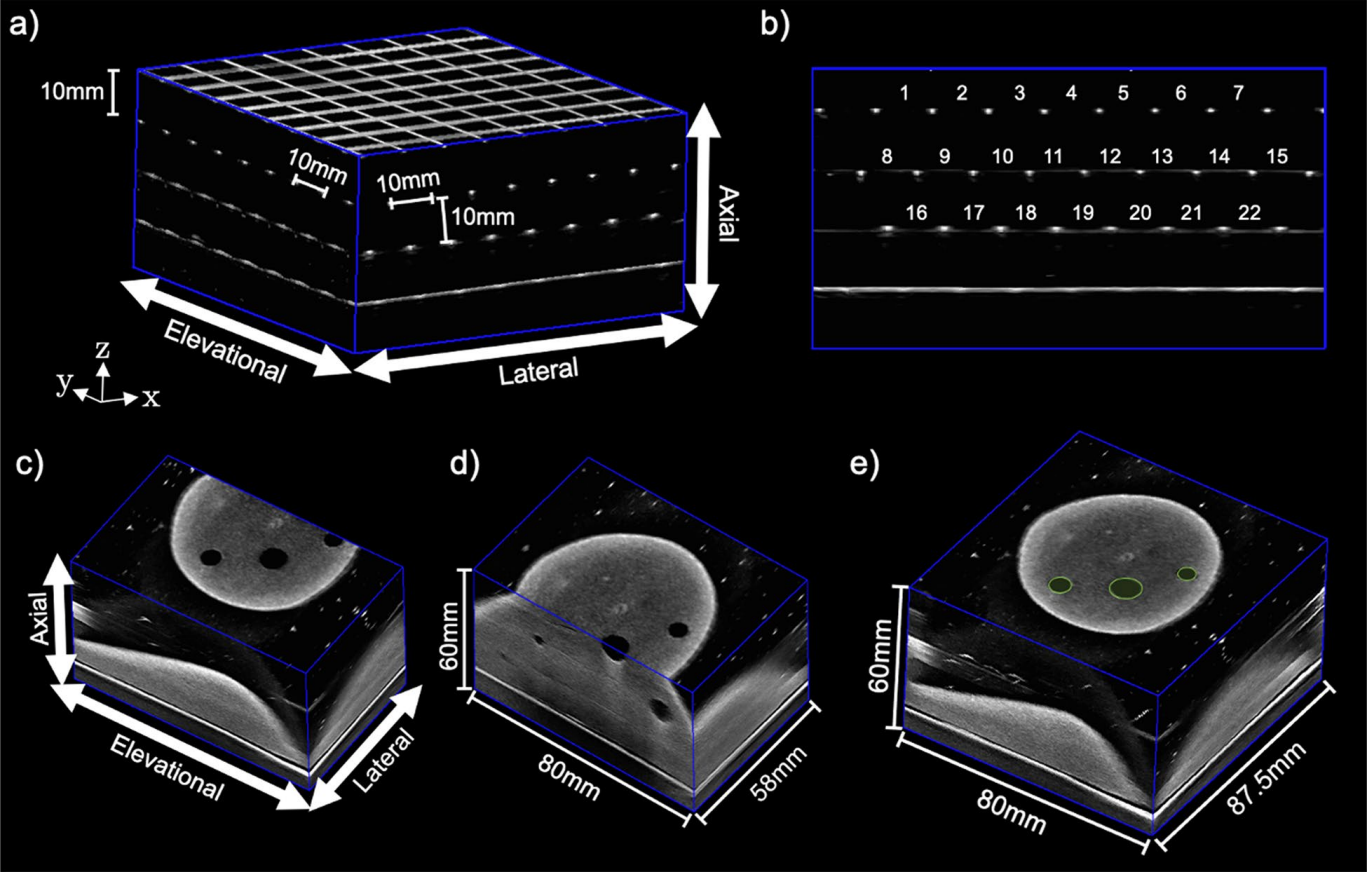
Top Row: Dedicated 3D ABUS images for geometric reconstruction validation in phantoms: (**a**) 3D grid phantom for linear distance measurements in the lateral, elevational, and axial US directions with an example measurement with nominal values 10.0 mm; and (**b**) sagittal plane with numbered lateral distance measurements. Bottom Row: 3D ABUS image in a tissue-mimicking breast phantom: (**c**) first-pass 3DUS image with the US directions, (**d**) second-pass 3DUS image after 29.5 cm lateral translation with the volumetric dimensions, and (**e**) combined 3DUS image with volumetric dimensions and indication of spherical inclusions 4.76 and 9.53 mm in diameter (as green contours) in the coronal view plane.

Linear reconstruction and measurement accuracy in each US image dimension was validated with a custom 3D grid phantom. The 3D grid phantom is made from crosshatched 0.1 mm diameter monofilament polyester wires with a 10.0 × 10.0 mm grid, positioned in four parallel layers spaced 10.0 mm apart in the elevational direction, as shown in Fig. 2a ^38^. The phantom was immersed in a solution made from a mixture of distilled water and 7.25% by volume isopropyl alcohol to mimic the approximate speed-of-sound in soft tissues of 1540 m s^−1^^39^. The 3D ABUS images were acquired at 25 frames s^−1^, 6 frames mm^−1^, 80 mm scan distance, 40 mm depth setting, and 29.5 mm lateral translation, resulting in a 3DUS image volume of 80 × 87.5 × 40 mm^3^ and voxel size of 0.14 × 0.14 × 0.33 mm^3^.

Linear accuracy measurements were obtained using in-house software workstation for visualization of the 3DUS images. To acquire linear measurements, the 3DUS image was visualized in the axial or sagittal view plane of interest. Linear reconstruction errors were computed by performing distance measurements (N = 22, each) between the center of two adjacent monofilament wires in the lateral, elevational, and axial (x, y, and z-axis) US directions, illustrated in Fig. 2a and b. The mean, standard deviation (SD), and upper and lower 95% confidence intervals of the error between the distance measurements and the expected nominal distance of 10.0 mm was computed. The intraobserver variability was computed for a single observer performing linear distance measurements between a pair of wires (N = 5) and computing the SD of the measurements for each lateral, elevational, and axial US directions.

The limits of the in-plane resolution (axial and lateral) of our 3D ABUS system were determined using a CIRS (CIRS, Arlington, VA, USA) model ATS539 phantom. Similarly, using a CIRS model 538NH phantom, the elevational resolution limits were examined.

#### 3D ABUS breast phantom studies and 3D reconstruction accuracy

An agar-based tissue-mimicking breast phantom (approximately 240 cm^3^ in volume) was fabricated with a mixture of 3% agar powder per mass, 8% glycerol per mass, and distilled water to mimic the speed-of-sound in soft tissues of approximately 1540 ms^−1^ ^39^. To simulate parenchymal breast tissues, 15% per mass SigmaCell cellulose scattering agent (MilliporeSigma, Burlington, Massachusetts, United States) was added to the background mixture. Agar-based small and medium spheres (4.76 and 9.53 mm in diameter) were fabricated with a 3D-printed template without added scattering agent and embedded into the breast phantom to appear hypoechoic relative to the background ‘tissue’ for identification and volumetric measurements.

To evaluate volumetric reconstruction and measurement accuracy, 3D ABUS images were acquired of the breast phantom. The 3D ABUS acquisition parameters were the same as for the 3D grid phantom experiments with an 80.0 mm scan distance, 60.0 mm depth setting, and 29.5 mm lateral translation. Figure 2 shows an example of a 3D ABUS image acquired of the breast phantom, where Fig. 2c and d show the two parallel acquired 3DUS images and Fig. 2e shows the combined 3DUS image of the phantom with its total volumetric dimensions and example of manually segmented spherical inclusions in the coronal plane.

Volumetric reconstruction was assessed by manually segmenting the spherical inclusions in each consecutive sagittal 2DUS image slices spaced 0.33 mm apart in the combined 3DUS image with 3D Slicer (3D Slicer, Version 4.11.0) in the Segment Editor module^40^. The mean, SD, and 95% confidence intervals of the spherical inclusion volumetric percent error (%) from its actual known volumes were computed with Eq. (1):

1 $$\%Error=\frac{\left|{V}_{1}-{V}_{2}\right|}{({V}_{1}+{V}_{2})/2 }\times 100\%$$

where ${V}_{1}$ is the nominal volume and ${V}_{2}$ is the measured volume of the spherical inclusions in the breast phantoms.

#### 3D ABUS imaging in healthy volunteer studies

**Figure 3:**
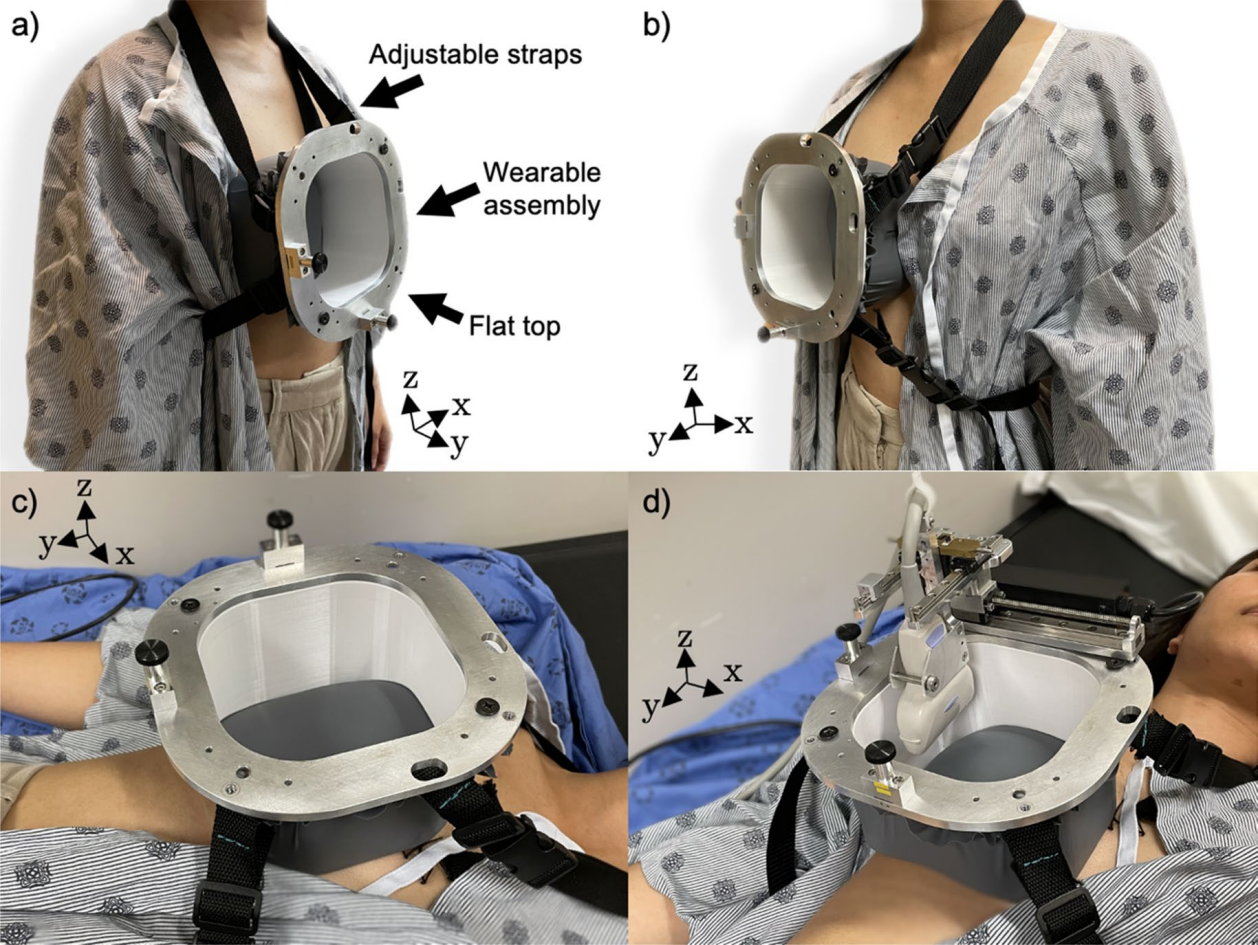
Images of the dedicated 3D ABUS system on a healthy female volunteer. Top Row: (**a**) 3D perspective view from a lateral camera position, showing the wearable 3D-printed dam assembly and disposable membrane attached onto the volunteer with the adjustable strapping mechanism; and (**b**) medial 3D perspective view from a medial camera position, showing that the contralateral breast is covered during acquisition. Bottom Row: Volunteer in supine position (**a**) without and (**d**) with the removable linear scanner for acquisition. Ultrasonic coupling gel would be placed inside of the dam and the ultrasound cradle would be adjusted prior to performing image acquisition.

The utility of the dedicated 3D ABUS system was evaluated in three healthy volunteers with written informed consent and the study was approved by the Western University (London, Ontario, Canada) Health Sciences Research Ethics Board (HSREB #114,347) at the Schulich School of Medicine and Dentistry. All healthy volunteer studies and methods were performed in accordance with the relevant guidelines and regulations of the HSREB. A depiction of the dedicated 3D ABUS system positioned on a healthy female volunteer is shown in Fig. 3. Informed consent was obtained from the healthy female volunteer to publish the images in an online open-access publication.

To evaluate our 3D ABUS approach across a broad range of diverse patient geometries and breast sizes, we imaged one healthy male volunteer (24 years of age, 22.0 BMI) and two healthy female volunteers, one with a 34A breast cup size (26 years of age, 22.0 BMI) and the second with a 32F breast cup size (27 years of age, 25.0 BMI). The healthy volunteers also provided informal feedback to evaluate the 3D ABUS system, specifically regarding patient comfort and functionality. Comfort evaluation included the physical ease of wearing the dam assembly, including the shape of the curvilinear dam base and the flexible tubing material. Functionality assessment included evaluating the effectiveness of the system in conforming to the natural curvature and shape of various breast sizes and geometries, allowing for the stabilization of the breast during 3D ABUS image acquisition.

The proposed clinical workflow to perform 3D ABUS imaging in a healthy volunteers or patients, including preparation, patient positioning, image acquisition, and cleanup was as follows:

1. A replaceable sonolucent membrane was attached onto the wearable dam assembly, then a US lotion was applied on the skin-facing surface for coupling.
2. The wearable dam assembly, as the base of the dedicated 3D ABUS system, was affixed to the patient over either the left or right breast in a comfortable, sitting or standing position. The nylon webbing straps were fastened around the patient, such that the dam assembly was positioned securely on the breast.
3. The patient lay supine, then low viscosity US transmission gel was poured into the dam assembly to cover the interior volume of the dam over the breast.
4. The optional compression assembly was positioned on the dam assembly. The knob was manually adjusted to lower the TPX compression plate to immobilize the breast with a desired compression level, without painful application.
5. The motorized 3D ABUS scanner was rigidly mounted on the top plate with the locating pins and stabilization magnets in either the (a) craniocaudal direction to acquire transverse 2DUS images or (b) mediolateral direction to acquire sagittal 2DUS images. This setup process from Step 1 to 5 takes approximately 10-min.
6. The US acquisition parameters, including the scan distance, lateral translation, depth setting, frame density, and frame rates were adjusted by a trained operator.
7. Computer-driven 3D ABUS image acquisition was performed by activating the motorized linear scanner with a simple button in the software workstation. The US cradle was translated laterally to a second preset position(s) to expand the volumetric field-of-view for whole-breast coverage. The scanning time for a single pass is up to 20-s, depending on the acquisition parameters, with a total maximum acquisition time of 40-s. The repositioning of the US cradle to another position is immediate and takes approximately one second.
8. The parallel and partially overlapping 3DUS image(s) were registered in real-time for bedside or offline whole-breast assessment with multiplanar 3D views in axial, sagittal, and coronal view planes, or oblique planes.
9. The cleanup involves the removal of the motorized scanner and dam assembly, entirely containing the ultrasonic coupling gel, and wiping off any remaining sonolucent lotion from the breast. This process takes approximately one-minute.

A wearable 3D-printed dam assembly with an approximately 1-inch wall at its maximum curvature height was used for the healthy male volunteer and healthy female volunteer with the 34A breast size. A second 3D-printed dam assembly with a 3-inch wall was used for the second healthy female volunteer with a 32F breast size. Feedback from the volunteers highlighted aspects of the 3D ABUS system that required adjustment for enhancing comfortability and functionality, as discussed in later sections of this work. After modifications were implemented, the volunteers all self-reported that the dam fit comfortably against their chest wall, with no leakage of US gel outside of the dam assembly. All 3D ABUS images were acquired at 25 frames s^−1^, 4 frames mm^−1^, 80.0–90.0 mm scan distance, and 40.0–60.0 mm depth setting, resulting in a 3DUS image volume of 80.0–90.0 × 87.5 × 40.0–60.0 mm^3^ and voxel size of 0.14 × 0.14 × 0.33 mm^3^. The total time to acquire a 3D ABUS image with these parameters was approximately 20–28 s: 10–14 s per each pass. In addition to acquiring dedicated 3D ABUS images of the healthy volunteers, the following parameters and acquisition variables were evaluated for their impact on 3D ABUS image quality: (a) use of the sonolucent membrane, (b) use of the compression assembly, and (c) effect of patient breathing and motion.

#### Sonolucent membrane

The 3D ABUS images were acquired with and without the use of the sonolucent membrane, as described in Step (1), to evaluate the impact on image quality. With the sonolucent membrane, US lotion was used to couple the membrane with the breast prior to affixing the 3D ABUS device. Standard low-viscosity US transmission gel was used to fill the volume inside the dam assembly in both cases. The optional compression assembly was not included in either case.

#### Compression assembly

3D ABUS images were acquired with and without the use of the optional compression assembly, as described in Step (4). With the compression assembly, minimal compression was used to immobilize the breast (without painful application) to distribute the breast tissues across the acquired volumetric field-of-view. The 3D ABUS image quality was evaluated by a trained observer, and patient comfort or discomfort was qualitatively reported by the healthy female volunteer.

#### Breathing and patient motion

The effect of patient breathing was assessed by acquiring 3D ABUS images with (a) breath-hold during 3D ABUS image acquisition and (b) deep-breathing from maximum inspiration to maximum expiration during 3D ABUS acquisition. Both experiments were performed without the compression assembly, as described in Step (4). The effect of patient motion was further assessed by instructing the volunteer to actively talk during the 3D ABUS image acquisition, representing a high level of motion in clinical practice.

## Results

### Geometric 3D ABUS reconstruction accuracy and breast phantom studies

The mean and SD linear distance measurement errors, and 95% confidence intervals compared with the nominal measurements (10.0 mm) for lateral, elevational, and axial US directions (N = 22, each) are summarized in Table 1. The mean distance errors ranged from 0.06 to 0.15 mm from the nominal measurements, with a maximum upper-limit error within 95% confidence of 0.18 mm in the axial direction (Table 1). The intraobserver variability between linear distance measurements ranged from 0.05 to 0.07 mm across the lateral, elevational, and axial directions, which are summarized in Table 1. The in-plane resolution (axial and lateral) of our proposed 3D ABUS system resulted in the ability of our system to resolve the smallest separation of wires of 1.0 mm in the axial direction and 2.0 mm in the lateral direction in the 3DUS image. The elevational resolution was found to be at a minimum of 1.5 mm at a depth of 12 mm.

**Table 1:** Summary of linear measurement errors and intraobserver variability in lateral, elevational, and axial US directions in the 3D grid phantom.

Linear measurements	Measurement direction		
	Lateral	Elevational	Axial
Nominal distance (mm)	10.0	10.0	10.0
Mean distance (mm)	10.06	10.05	10.15
Mean error (mm)	0.06	0.05	0.15
Standard deviation (mm)	0.09	0.06	0.07
95% confidence interval (mm)	0.03, 0.10	0.02, 0.07	0.12, 0.18
N	22	22	22
Intraobserver variability			
Standard deviation (mm)	0.06	0.07	0.05
N	5	5	5

The volumetric reconstruction and measurements of the small and medium sized spherical inclusions (4.76 mm and 9.53 mm in diameter) in the tissue-mimicking breast phantom are summarized in Table 2. The mean volume percent errors and SD for the small and medium spherical inclusions (N = 5, each) were 1.26 ± 1.26 mm^3^ and 9.44 ± 7.84 mm^3^, which corresponded to a percent difference of 2.23% and 2.11%, respectively. The overall volumetric percent error across all spherical inclusions in the breast phantom was 2.18%.

**Table 2:** Summary of volumetric measurement errors of the spherical inclusions in the tissue-mimicking breast phantom.

Volumetric measurements	Spherical inclusions		
	Small	Medium	Both
Spherical diameter (cm)	4.76	9.53	–
Nominal volume (mm^3^)	56.47	453.19	–
Mean measured volume (mm^3^)	57.51	443.74	–
Mean volume error (mm^3^)	1.26	9.44	–
Standard Deviation (mm^3^)	1.26	7.84	–
Mean % difference	2.23%	2.11%	2.18%
N	5	5	10

### 3D ABUS in healthy volunteer studies

The reports from our healthy volunteer focus group initiated some changes in our system design, specifically the wearable dam assembly. First, we adjusted the curvilinear shape of the patient dam to enhance its conformity to the natural curvature of the chest wall, while accommodating various breast sizes and shapes. Volunteers indicated that the medial side of the dam, which runs parallel to and positioned on the sternum, should be slightly shallower compared to the lateral side. This adjustment is intended to account for potential variations in the chest wall’s natural shape and to optimize the fit of the dam for both breasts. Additionally, the flexible tubing on the base of the dam assembly should be made from a softer material for improved conformity across different body types, allowing for more flexibility between different shapes and variations between breasts. In response to this feedback, the dam assembly underwent minor refinements to modify the curvilinear base dimensions and to include a softer neoprene tubing material. In addition, volunteer feedback provided insights on the adjustability of the dam assembly, specifically pertaining to the straps and fastening mechanism. Alterations were made to include adjustable mechanisms on the strap webbing and the fastening mechanism, allowing for quick, secure, and modifiable attachment of the dam assembly to the volunteer. Feedback from volunteers after the dam assembly alterations indicated that the overall user experience was improved, and initial discomfort of the wearable device was alleviated. This optimized dam assembly was utilized in all three healthy volunteers, with adjustments to the maximum height to encompass breast size.

**Figure 4:**
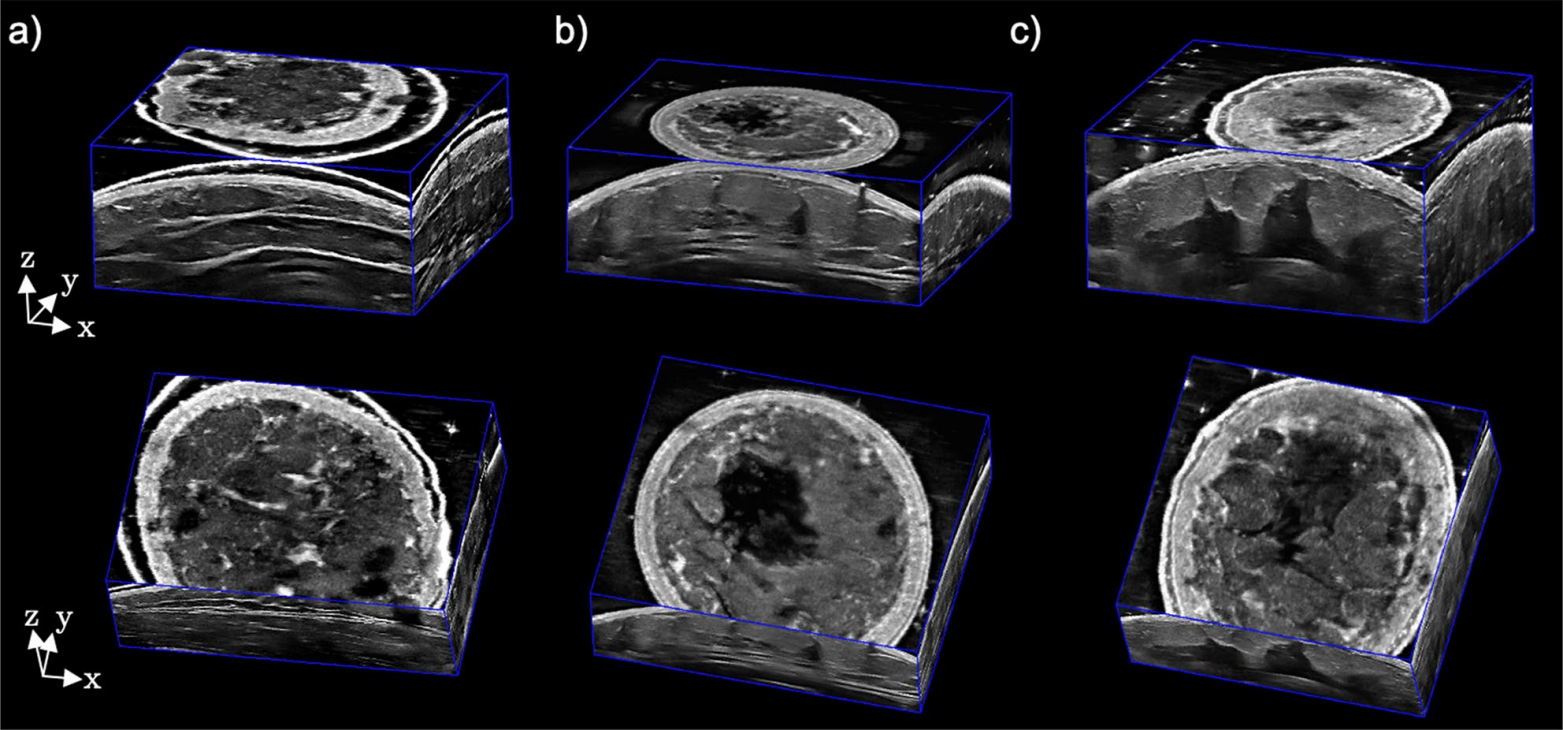
Dedicated 3D ABUS images in healthy volunteers: (**a**) male volunteer, (**b**) female volunteer with a 34A breast size, and (**c**) healthy female volunteer with a 32F breast size in 3D perspective and coronal views.

The 3D ABUS images acquired in the healthy male and healthy female (34A and 32F) volunteers are shown in Figs. 4a–c, respectively. The 3D ABUS images are shown in multiplanar 3D views (sagittal, axial, and coronal view planes) for whole-breast evaluation and assessment. Additionally, the 3D ABUS images can also be viewed in any oblique plane, where the user can dynamically slice the volume in any direction and orientation in real-time. Anatomical structures of the male and female breast, including the skin, nipple, areola, subcutaneous fat lobules, subcutaneous fat layer, glandular tissues (ducts and lobules), Cooper’s ligaments, pectoralis muscles, pleura, ribs, intercostal muscles, and lungs were identified in the 3D ABUS images^41^. These anatomical features and details are clearly visualized as distinct layers in the 3DUS images based on characteristic differences in sonographic appearance from hypoechoic (i.e., fatty tissues and ducts) to hyperechoic (i.e., fibrous tissues and Cooper’s ligaments) structures^41, 42^. All volunteer 3D ABUS images show the breast in a natural, supine anatomical position since there is no compression or transducer pressure required when acquiring the images, unlike with conventional HHUS or commercial ABUS applications.

**Figure 5:**
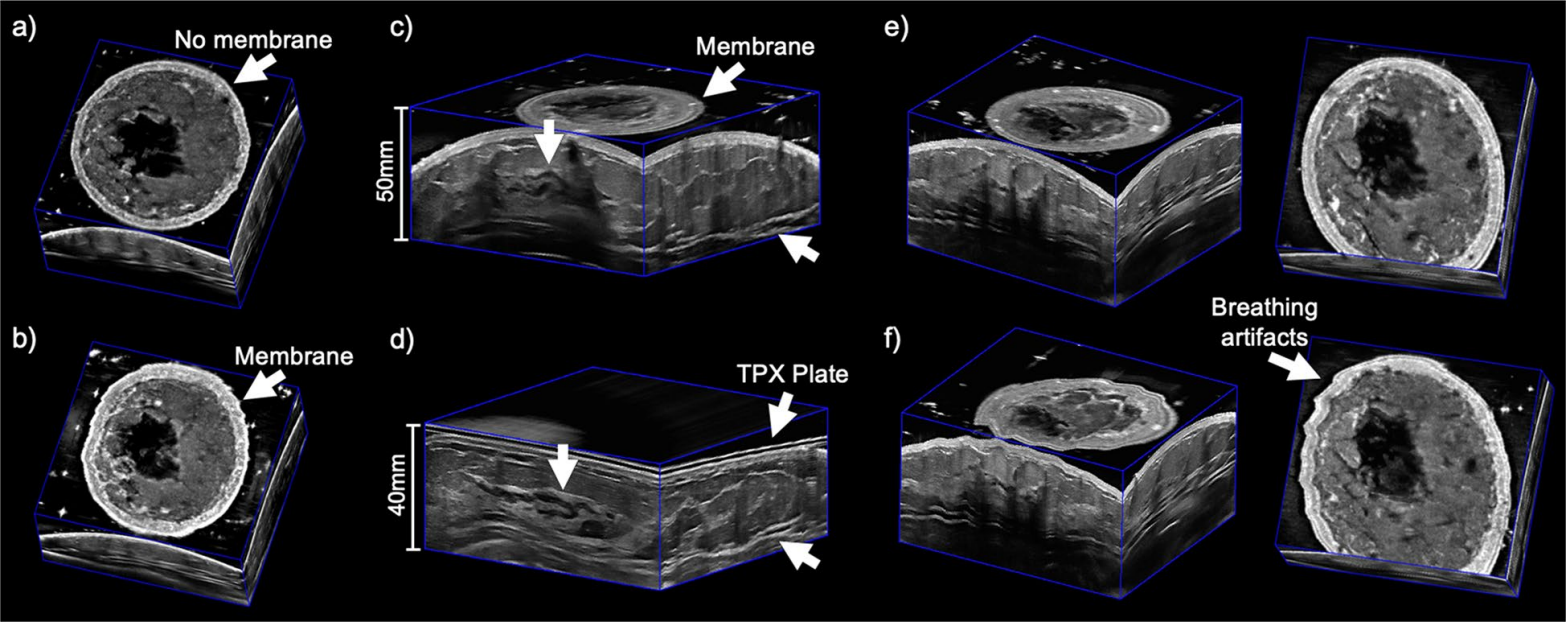
Dedicated 3D ABUS images: (**a**) without and (**b**) with the sonolucent membrane, which appears as a hyperechoic contour above the skin in the coronal view; (**c**) without and (**d**) with the compression assembly, white arrows indicate corresponding anatomical features in the uncompressed and compressed images; and (**e**) with breath-hold and (**f**) deep-breathing from complete inspiration to expiration during 3D ABUS acquisition in 3D perspective and coronal views, white arrows indicate anatomical features (hyperechoic skin) that are slightly distorted due to cyclic breathing.

3D ABUS images acquired with and without the use of the sonolucent membrane are shown in Fig. 5a and b, respectively. While the internal anatomy remains consistent, there is a clear hyperechoic contour of the taut membrane above the skin (Fig. 5b). Across the three healthy volunteers, slight differences are observed in the positioning of the membrane relative to the skin, which can be attributed to an excessive amount of US lotion as a hypoechoic layer between the skin and membrane. This is clearly visualized in the male volunteer, where there is a hypoechoic layer of coupling gel between the skin and hyperechoic membrane (Fig. 4a). The use of the sonolucent membrane to immobilize the breast was reported as very comfortable by all three volunteers. Since the sonolucent membrane entirely covered the entire breast, once the 3D ABUS assembly was affixed to the volunteer, the breast was not exposed during the entire image acquisition process, allowing improved comfort and modesty during acquisition. Moreover, the membrane prevented the direct contact of US gel with the skin surface, allow for simpler cleanup after image acquisition.

Figure 5c and d shows 3D ABUS images acquired without and with the compression assembly. 3D ABUS images without the compression assembly maintains the natural shape and curvature of the breast (Fig. 5c). With the compression assembly, there was an improved distribution of the breast tissues and reduction in US penetration depth, from 50.0 mm and 40.0 mm US depth settings, which improved the quality of the anatomical features and details in the images while avoiding structural distortions due to excessive compression. Furthermore, there was a visible reduction of reverberation artifacts due to air bubbles in the coupling material due to improved coupling with the compression plate. However, there were reverberation artifacts observed in the 3D ABUS image with the compression assembly due to the properties of the TPX plate, as shown in Fig. 5c.

**Figure 6:**
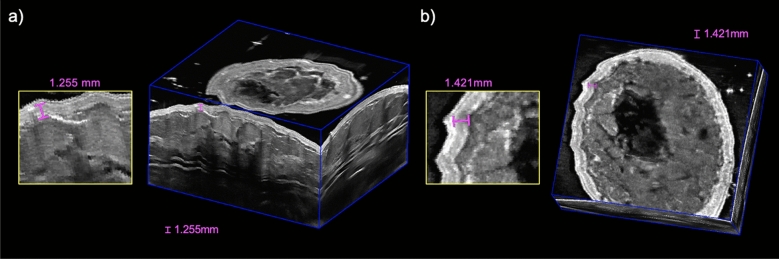
3D ABUS images acquired with deep-breathing from complete inspiration to expiration during image acquisition in (**a**) 3D perspective and (**b**) coronal views showing slight distortions, with an example measurement of the maximum displacement of 1.26 mm and 1.42 mm, as shown in the hyperechoic skin due to cyclic breathing.

3D ABUS images were further acquired in a healthy female volunteer with a breath-hold and while deep-breathing from a complete inspiration to expiration during acquisition, as an extreme case of breathing and motion, shown in Fig. 5e and f in 3D perspective and coronal views, respectively. Deep breathing during 3D ABUS image acquisition resulted in small cyclic motion artifacts in the skin and internal anatomical structures, which is consistent with the healthy volunteer’s complete inspiration and expiration, indicated as white arrows in Fig. 5f. These artifacts resulted in distortions that were approximately 1.26 mm and 1.42 mm in the sagittal and coronal view planes, which propagated as small smears in the coronal direction, as shown in Fig. 6a and b, respectively. No other observable artifacts were found to impact the quality of the images. The artifacts seen in the depicted 3D ABUS image represented an extreme case of breathing, while natural free-breathing was employed during the 3D ABUS acquisitions in the other healthy volunteer studies without any substantial artifacts, as shown in Fig. 4.

## Discussion

We developed, validated, and investigated the potential utility of a dedicated 3D ABUS system. Our 3D ABUS system exemplifies the advantages of ABUS systems, enabling standardized acquisition and potentially reduced operator-dependence and increased time efficiency, and volumetric 3D visualization for whole-breast assessment^28^. Beyond these advantages, there are several key design features of our proposed 3D ABUS system that overcome some limitations of alternative ABUS systems. Commercially available systems are typically costly and bulky, as they require a complete dedicated system and workstation, including articulated manipulators, specialized US transducers^18, 31, 32^, or specialized patient examination tables for prone acquisition^28, 34^, which limit its overall ease of portability. An alternative, emerging approach that addresses some of these challenges is the ATUSA system (iSonoHealth, California, United States), which allows for portable ABUS image acquisition with a compact wearable accessory with an integrated specialized US scanning mechanism^43^. However, our dedicated 3D ABUS system is cost-effective, adaptable, and versatile. Specifically, our system is fabricated from entirely economical materials and utilizes rapid-prototyping and 3D-printing to accommodate any commercially available US machine and transducer. This maximizes the ease of integration of the dedicated 3D ABUS system into most existing clinical settings, which includes limited-resource or cost-constrained settings, as US imaging is a widely available and accessible modality in clinical practice. When considering settings without availability to US machines, the 3D ABUS system can be adapted to accommodate transducers with portable US machines, which would further improve its transportability and point-of-care applicability.

In addition, the dedicated 3D ABUS system is a portable unit that removes the need for external components, unlike most commercially available ABUS systems. Moreover, its constituent components (i.e., dam assembly, compression assembly, and motorized 3DUS scanner) are compact and its assembly is relatively intuitive, since all components can be easily attached or removed from the wearable dam assembly that conforms to the patient and serves as the base. Since the 3D ABUS acquisition approach utilizes a supine positioning approach, the natural shape and curvature of the breast is maintained, potentially allowing for correlation with alternative imaging modalities, such as CT or MRI, when similarly acquired in a supine position. Furthermore, the 3D ABUS system is a patient-specific device, as its wearable base assembly can be personalized to conform to the patient or specific patient populations. Specifically, its curvilinear base can be adapted with rapid-prototyping and the flexible, skin-safe Neoprene tube on the underside of the dam (i.e., patient contact side) to accommodate diverse populations, including variable patient geometries, and sizes. This design consideration potentially opens doors for more inclusive practices to advance gender-affirming care in sexual and gender diverse populations. Importantly, another advantage of our proposed 3D ABUS design is the ability to acquire and visualize whole-breast images within a single 3D volume. In comparison with alternative, existing commercially available systems, multiple scans at various angles and geometries are required to encompass the entity of the breast. Our proposed system, instead, places the entire breast in a natural position within the dam assembly, improving image coverage during acquisition. The portability, cost-effectiveness, and patient-specific features of the 3D ABUS system allows for utility across various settings, including limited-resource or underserved populations, where advanced technologies or techniques may not be easily accessible.

In addition to the realization and fabrication of the 3D ABUS system, we experimentally validated its geometric reconstruction accuracy with various phantoms. With a 3D grid phantom, the linear measurement errors showed excellent accuracy and reliability. While there were no substantial differences between the linear measurement directions in the 3D ABUS image, the maximum error in the axial (z-axis) direction may be attributed to the increased difficulty to discriminate the central axis of the wires due to the deterioration of axial resolution from the focal zone of the US beam. Additionally, there were small reverberation artifacts in the monofilament wires, which may have accounted for some uncertainty in the linear measurement errors. While the linear measurements demonstrated excellent accuracy and reliability, it should be noted that these measurements were susceptible to potential bias, since the observer performed linear measurements with a software module that displayed the distances in real-time. This may introduce perception biases and overestimate the accuracy and precision of the linear distance measurements. 3D ABUS imaging was also demonstrated in a tissue-mimicking breast phantom. The small volumetric reconstruction error of the spherical inclusions in the acquired 3D ABUS images are consistent with previously validated mechanically-driven 3DUS acquisition approaches^44, 45^. Since the volumetric measurements were blinded to the observer prior to comparison with their known volumes, potential biases were minimized, increasing the reliability and validity of this finding. Geometric reconstruction accuracy is necessary to enable reliable quantitative breast lesion measurements and volumetric estimation in clinical practice, which are important diagnostic and prognostic factors in breast cancer management^46^. Moreover, accurate 3D measurements in axial, sagittal, and coronal view planes may allow for increased accuracy in clinical applications, overcoming limitations associated with operator-dependent HHUS^46^.

The 3D ABUS system and its proposed clinical workflow was demonstrated in three healthy volunteers, as a proof-of-concept toward bench-to-bedside translation for breast cancer screening and diagnostic applications. The three recruited healthy volunteers represented a broad range of diverse patient geometries, shapes, and sizes. Feedback from the healthy volunteer focus group indicated critical system requirements for improved user experience and reduced discomfort, which would translate to critical patient requirements when implemented into clinical practice. Additional requirements that will be evaluated in a future study include whole-breast imaging coverage compared with gold-standard modalities, ease of use, and other clinical workflow considerations. Importantly, iterative modifications that were made to the system design to improve patient comfort and functionality in this study further inform the utility of the 3D ABUS system as a wearable design, compared with external imaging systems that may use painful compression techniques during image acquisition. Regarding the symmetry of the dam assembly for 3D ABUS acquisition, the current dam assembly can adequately be positioned on both breasts. However, to address the potential barrier of bilateral asymmetry, particularly with prospective 3D ABUS system designs involving a dam assembly on each breast to streamline workflow, further design refinements may be warranted to optimize the fit on both breasts. While the healthy volunteer feedback provided valuable insights for image acquisition, future studies are necessary to evaluate our prototype 3D ABUS system in a relevant clinical context.

3D ABUS imaging of the healthy volunteers showed high-resolution whole-breast images with clearly visualized anatomical structures and details. The healthy male volunteer depicted distinct conventionally masculine anatomical features, including a wider chest, which exemplified minimal breast curvature and tissue deformations with sparsely distributed glandular tissues and ducts^41^. The healthy female volunteers demonstrated a wide range of breast sizes (34A and 32F), representing substantial differences in breast tissue projection or expansion (bust measurement) from the chest wall (band measurement) of 1-inch and 6-inches, respectively. This demonstrated the capability of the proposed 3D ABUS approach to acquire images in both smaller and larger breasts (A–F breast cup sizes) with this proof-of-concept, with minimal modifications to the personalizable 3D-printed dam assembly. Across all three healthy volunteers, the proposed 3D ABUS imaging workflow (Section “3D ABUS imaging in healthy volunteer studies”) from preparation, patient setup, and image acquisition was feasible in practice. Moreover, this workflow was performed as a point-of-care approach, where the entire preparation, image acquisition, multiplanar 3D visualization and interpretation were performed at the bedside in a time-efficient manner, with 3D ABUS image acquisition taking approximately 20 s, allowing for immediate assessment of the 3D ABUS images.

High-quality 3D ABUS imaging with most commercially available systems are reliant on proper operator training for patient positioning and coupling with the breast to avoid contact artifacts, motion artifacts, and skip artifacts due to motion of some breast lesions during acquisition^31^. Our 3D ABUS system employs specific design features and functionalities to minimize these technical acquisition limitations. The sonolucent membrane provides a means to improve tissue stabilization, maintain contact, and immobilize the breast, as determined by the end-users of the system as well as reports from the healthy volunteer focus group. While use of the membrane was able to maintain image quality and did not cause any visible distortions to the anatomical structures, optimal use of the membrane requires minimizing the amount of coupling US lotion between the membrane and the breast to ensure the membrane was taut. One limitation of the use of the membrane was its difficulty to visually localize and assess the nipple position to ensure that the breast was centered in the volumetric field-of-view. Future work will evaluate the reproducibility of positioning the wearable assembly on various patients, and then assess the whole-breast volumetric coverage compared with MRI or alternative quantitative imaging methods. Moreover, comparison to conventional mammography or DBT has been proven useful in the literature^47–49^. Future studies will investigate correlating our acquired 3D ABUS images to those acquired in mammographic views with design modifications or deformable image registration approaches. Optimization of volumetric coverage, including the potential for optimized acquisition geometries, such as hybrid scanning approaches with adjustable angle of lateral incidence, may require modifications of the 3D-printed dam assembly shape, increasing the window size, or modifying the motorized scanner mechanism and automated pathway for image acquisition.

All 3D ABUS images of the healthy volunteers (Fig. 4) were acquired with natural free breathing and minimal artifacts were observed. When considering an extreme case of breathing and motion, one healthy volunteer performed deep-breathing with complete inspiration and exhalations during the 3D ABUS acquisition. Image outputs showing these cases are shown in Fig. 6. With deep breathing, there were minor artifacts observed, which presented only as small distortions in the scanning (elevational) direction, consistent with the breathing cycle. Deep breathing resulted in displacements of approximately 1.42 mm and 1.26 mm in the coronal and sagittal planes, respectively, in the skin and internal structures in the 3D ABUS image, which are smaller than chest displacements observed during free breathing of up to 3–5 mm^50^. This was possible, as the 3D ABUS system and its frame of reference were affixed directly onto the patient during image acquisition. Furthermore, optimization of the strapping mechanism (i.e., strap materials and placement) could further mitigate breathing and motion artifacts by moving synchronously with the patient as they breathe and move. Importantly, optimizing 3D ABUS image quality with free breathing would remove the dependence for complex breath-hold methods, further improving overall patient comfort and simplifying workflow in clinical practice.

The impact of the compression assembly on 3D ABUS acquisition and image quality was evaluated. The 3D ABUS acquisition performed without the compression assembly effectively removed any applied compression or pressure, while maintaining the natural curvature and structure of the breast. This approach could be especially useful for women who are lactating, where minimal compression is required when imaging sensitive structures and abnormalities in the breast^51^. However, in some cases, there was substantial nipple shadowing and attenuation in the central region of the breast, which resulted in an anechoic region in the 3D ABUS image, as shown in Fig. 5c. Acoustic shadowing behind the nipple is a common artifact in ABUS images, which results from heterogenous tissue distribution and anisotropic acoustic impedance of the nipple, which may distort the US beam, increase difficulty to interpret images, and obscure breast lesions^32, 51, 52^. Importantly, the compression assembly was able to mitigate these artifacts and improve image quality by better distributing the breast tissues, as shown in Fig. 5d. Specifically, the compression enabled the breast tissues and structures to be reoriented more perpendicular to the US beam with a reduction in required US penetration depth. This improved the discrimination of small anatomical structures and details, such as milk ducts, while avoiding geometric distortions due to excessive compression, as shown in Fig. 5d. While the image quality was improved with the compression assembly, there were reverberation artifacts observed due to the TPX plate. Future studies involve optimizing the material of the compression plate to minimize this artifact. Moreover, while low viscosity gel was utilized to mitigate air bubbles, air bubbles still became trapped beneath the TPX compression plate, which was a limitation to our current design. Future work is focused on improving the design of the TPX plate by strategically implementing holes into the periphery of the plate to allow for air bubbles trapped beneath the TPX plate to escape during gentle immobilization or compression. This proposed alteration would further reduce potential coupling artifacts due to air bubbles in the acquired 3D ABUS images. In our healthy volunteer feedback, the proposed compression assembly mechanism was reported as pain free by the healthy female volunteer, which may overcome limitations of commercially available ABUS systems with preset compression levels, where image acquisition has been reported as painful by patients^33^.

We proposed a 3D ABUS approach, which included several design, technical, and potential clinical workflow advantages, demonstrated through the system’s fabrication, experimental validation, and healthy volunteer studies; however, there are some limitations in this study and areas for future work. Although the 3D ABUS system can accommodate any commercially available US transducer, thereby improving its cost-effectiveness and versatility, its 3D spatial resolution is limited in scanning direction due poor elevational resolution of conventional linear array US transducers, as determined by the elevational beamwidth^53^. Therefore, the 3D ABUS image has a high in-plane resolution (i.e., high-resolution transverse or sagittal US images for craniocaudal and mediolateral orientations, respectively) and poor out-of-plane resolution in the scanning direction, which is illustrated in Fig. 5. Future studies will focus on improving the 3D resolution in the 3D ABUS image with a 3D complementary breast (CB) US approach^54^. Another limitation is the current accessibility and adaptability of our prototype 3D ABUS design for widespread adoption in clinical practice, despite its cost-effectiveness and versatility. Adapting our SolidWorks CAD models to open-source programs would allow for improved dissemination for 3D ABUS design development, alteration, personalization for diverse patient populations, and optimization across various settings.

While our study demonstrated adaptability of our design, geometric 3D reconstruction accuracy, 3D ABUS imaging capability in volunteer studies, and ability to mitigate motion artifacts, future extensive studies are still required to demonstrate its robustness across various diverse patient populations, including those with dense breasts. Prior to this exploration, further investigation is required to examine the robustness and adaptability of the proposed 3D ABUS system across a wider range of breast sizes and geometries that would be expected in clinical practice. Since this study only included healthy volunteer studies, its utility will be explored in a clinical feasibility study in comparison with supplemental HHUS, commercial ABUS systems, and screening mammography. Moreover, and of substantial importance, the overall impact that the proposed 3D ABUS system has on current clinical practices and workflow in breast cancer screening will be qualified in a future study. This includes an examination of the detection capability of our proposed 3D ABUS system, especially when compared to commercial systems used in the same context. With the reported results from this current study, a clinical feasibility with patients will be performed with a separate Research Ethics Board (REB) and Canadian regulatory (Health Canada) approval. Furthermore, its operator and device agnosticism will be evaluated to assess its ability to be implemented as a point-of-care screening approach in limited-resource or cost-constrained settings. This is especially important when considering environments with limited healthcare operator expertise, where high-quality image acquisition could provide reliable offline or remote assessment and interpretation for screening or diagnostic applications.

## Conclusions

We developed a cost-effective, portable, patient-dedicated 3D ABUS system for point-of-care breast cancer screening, which was fabricated with economical materials, can accommodate any commercially available US machine and transducer, and is personalizable to diverse patient populations. Our proposed system is especially advantageous due to its ability to provide full image coverage of the entire breast in a single 3D volume. Geometric reconstruction validation in phantoms demonstrated accurate linear and volumetric measurements, showing potential for quantitative measurements for screening and diagnostic evaluation. Demonstration of the 3D ABUS system and proposed workflow in three healthy volunteers showed utility for point-of-care 3D ABUS image acquisition, and whole-breast visualization and assessment. Importantly, the wearable assembly components enable improved tissue stabilization and comfort, adjustable compression, and mitigates potential acquisition artifacts due to patient motion and breathing since its frame of reference is affixed directly onto the patient. While future studies are required to evaluate its robustness across diverse patient populations and acquisition conditions, the 3D ABUS system shows potential utility for widespread adoption into clinical care for accurate, robust, and effective breast cancer screening applications, especially in increased-risk populations and limited-resource settings.

## Data Availability

The data that support the findings of this current study are available from the corresponding author upon reasonable request.
